# Children With Diabetes and At Least One Non-Autoimmune Feature Should Be Considered for Monogenic Diabetes Testing

**DOI:** 10.1210/clinem/dgaf430

**Published:** 2025-08-01

**Authors:** Rebecca Myers, Melek Yildiz, Mehmet Nuri Ozbek, Jaida Manzoor, Mohsina Ibrahim, Chittaranjan Yajnik, Muge Atar, Zeynep Şiklar, Sezer Acar, Evgenia Globa, Omneya Magdy Omar, Huseyin Demirbilek, Samar Hassan, Korcan Demir, Misbah Hanif, Tulay Guran, Nihal Hatipoglu, Cemil Koçyiğit, Kevin Colclough, Jayne Houghton, Andrew Hattersley, Rachel Van Heugten, Kashyap Patel, Monogenic Diabetes Consortium, Y Abdelmeguid, S Abourazzak, A Annamalai, E Bhowmik, G Catli, S Chapman, H Eideh, V Jain, T Kontbay, V Mulliqi Kotori, G Supriya, S Musa, M Šandrk Beslać, M Sharaf, J Yong, G Yesiltepe Mutlu, R Yildirim, O Yilmaz, M Berberoglu, T Akcay, C Datar, S Dhadge, K Jog

**Affiliations:** Institute of Biomedical and Clinical Science, University of Exeter, Exeter EX2 5DW, UK; Department of Pediatric Endocrinology, Istanbul University Faculty of Medicine, 34093 Istanbul, Turkey; Department of Paediatric Endocrinology, Gazi Yasargil Diyarbakir Training and Research Hospital, 21100 Diyarbakir, Turkey; Department of Pediatric Endocrinology and Diabetes, Children's Hospital, University of Child Health Sciences, Lahore 54600, Pakistan; National Institute of Child Health, Paediatric Endocrinology and Diabetes Department, Karachi 75510, Pakistan; Department of Diabetes, King Edward Memorial Hospital & Research Centre, Pune 411011, India; Pediatric Endocrinology, Antalya Training and Research Hospital, 7100 Antalya, Turkey; Pediatric Endocrinology, Ankara University School of Medicine, 6100 Ankara, Turkey; Division of Paediatric Endocrinology, Dr.Behcet Uz Child Disease and Paediatric Surgery Training and Research Hospital, 35210 Izmir, Turkey; Department of Pediatric Endocrinology, Ukrainian Scientific and Practical Center of Endocrine Surgery, Transplantation of Endocrine Organs and Tissues, MoH of Ukraine, Kyiv 1021, Ukraine; Faculty of Medicine, Alexandria University, Pediatrics, Alexandria 21321, Egypt; Faculty of Medicine, Paediatric Endocrinology, Hacettepe University, 6230 Ankara, Turkey; Department of Pediatric Endocrinology, Gaafar Ibn Auf Pediatric Tertiary Hospital, Khartoum 11111, Sudan; Department of Pediatric Endocrinology, Dokuz Eylül University, 35340 Izmir, Turkey; Dow University of Health Sciences, Clinical Genetics Pediatrics, Karachi 75600, Pakistan; Department of Pediatric Endocrinology and Diabetes, Marmara University Hospital, 34841 Istanbul, Turkey; Department of Paediatric Endocrinology, Erciyes University, School of Medicine, 38039 Kayseri, Turkey; Department of Pediatric Endocrinology, Izmir Tepecik Training and Research Hospital, 35250 Izmir, Turkey; Exeter NHS Genomics Laboratory, Royal Devon University Healthcare NHS Foundation Trust, Exeter EX2 5DW, UK; Exeter NHS Genomics Laboratory, Royal Devon University Healthcare NHS Foundation Trust, Exeter EX2 5DW, UK; Institute of Biomedical and Clinical Science, University of Exeter, Exeter EX2 5DW, UK; Exeter NHS Genomics Laboratory, Royal Devon University Healthcare NHS Foundation Trust, Exeter EX2 5DW, UK; Institute of Biomedical and Clinical Science, University of Exeter, Exeter EX2 5DW, UK

**Keywords:** diabetes, monogenic diabetes, pediatrics, syndromic diabetes, genetic testing

## Abstract

**Context:**

Monogenic diabetes testing in children currently targets maturity onset diabetes in the young (MODY) or recognized genetic syndromes.

**Objective:**

We aim to determine whether genetic testing for monogenic diabetes should be performed for all children with diabetes and at least one non-autoimmune extra-pancreatic feature (syndromic diabetes).

**Methods:**

We recruited 183 children with diabetes and at least one non-autoimmune extra-pancreatic feature (50% [n = 91] with self-reported consanguinity). We measured islet-autoantibodies and type 1 diabetes (T1D) genetic risk score (T1DGRS) and used targeted next-generation sequencing to analyze all known causes of monogenic diabetes.

**Results:**

Of the children, 33% (61/183) had confirmed monogenic diabetes. Of these, 84% (51/61) had recessive etiologies with variants in *WFS1* (46%), *SLC19A2* (12%) and *SLC29A3* (12%) being most common. Monogenic cases compared to non-monogenic had similar age of diagnosis (7.4 vs 6, *P* = .1) and body mass index z-score (−0.08 vs −0.41, *P* = .3) but had higher parental consanguinity (62% vs 19%, *P* = .01) and features in multiple organ systems (53% vs 28%, *P* = .01). Only 59% reported well-recognized features of their associated genetic syndrome. Children with low T1DGRS (<50th centile of T1D population) and negative/untested antibodies were more likely to have monogenic cause compared to positive antibodies or negative/untested antibodies and a high T1DGRS (≥ 50th centile) (48% vs 3% vs 7%, *P* < .0001).

**Conclusion:**

Children with diabetes and at least one non-autoimmune extra-pancreatic feature should be considered for monogenic diabetes testing. Measurement of islet-autoantibodies and T1DGRS help prioritize genetic testing.

Establishing a monogenic diagnosis for diabetes with non-autoimmune extra-pancreatic features (syndromic diabetes) is crucial. It can guide targeted treatment (eg, thiamine for thiamine-responsive megaloblastic anemia syndrome), provide prognostic insights (eg, predicting neurological feature onset in Wolfram syndrome), and guide management of associated complications (eg, cardiomyopathy in maternally-inherited diabetes and deafness [MIDD]) (1-3). While some forms of monogenic diabetes present as isolated diabetes in early adulthood—for example, maturity onset diabetes in the young (MODY) subtypes HNF1A, HNF4A, and GCK (4)—others present as well-described diabetes syndromes. Importantly, the clinical presentation of monogenic syndromic diabetes varied; recent studies found 17.9% to 19% of suspected MODY cases harbored pathogenic variants in syndromic diabetes genes (3, 5). Physicians also face diagnostic challenges due to the ongoing discovery of novel monogenic diabetes subtypes (6, 7). There is also limited research into recessively inherited diabetes syndromes, as they are uncommon and frequently isolated in highly consanguineous populations that are under-researched in our current clinical genetics literature. These factors collectively contribute to the underdiagnosis of monogenic diabetes syndromes.

Undertaking genetic testing of children with diabetes and at least one non-autoimmune feature, irrespective of type or severity, may address this. However, it may cause excessive testing in consanguineous populations with a high burden of recessively inherited disorders where additional clinical features could be coincidental and unrelated to the underlying diabetes etiology (8). We aim to assess whether children with diabetes and at least one non-autoimmune extra-pancreatic feature should undergo comprehensive genetic testing and evaluate if clinical features, islet-autoantibodies, and type 1 diabetes genetic risk score (T1DGRS) can refine selection for genetic testing.

## Research Design and Methods

### Study Population

We recruited 183 unrelated probands from diabetes clinics between May 2016 and October 2023, focusing on populations with high consanguinity (9-12). Participants were included with diabetes onset between 9 months and 18 years and at least one non-autoimmune extra-pancreatic feature. In Turkey, 68 children were recruited as part of the GOOD study (13). All participants parents and guardians gave informed consent for genetic studies approved by the North Wales ethics committee (no. 17/WA/0327).

### Genetic Testing

For genetic testing, DNA extracted from EDTA blood samples collected between May 2016 and October 2023. We performed targeted next-generation sequencing of genes known to cause dominant and recessive monogenic diabetes subtypes (13). The detailed analysis methods used are described in Supplementary Material (14). For patients with pathogenic variants in syndromic diabetes genes, we analyzed whether cases exhibited a recognized clinical phenotype associated with the expected syndrome. Additional features were deemed recognizable if found in more than 50% of individuals with the described genetic cause or reported as common in published review articles (Table S1) (14).

### Biomarkers

Islet-autoantibody testing results for glutamic acid decarboxylase (GAD), insulin antigen-2 (IA-2), and zinc transporter-8 (ZnT8) antibodies were available for 58% of children (106/183). Where serum samples were available (77/106), testing was performed by Exeter Biochemistry laboratory using our well-described assay (15). Where serum samples were unavailable (29/106), clinician-reported islet-autoantibody status was used.

We generated a weighted type 1 diabetes genetic risk score (T1DGRS) from the 10 highest weighted T1D–associated single nucleotide polymorphisms (16). We calculated T1DGRS centiles using 1963 gold-standard T1D patients from the Wellcome Trust Case Control Consortium (WTCCC) as a reference population (17).

### Statistical Analysis

We compared categorical and continuous variables using the Fisher Exact and Mann-Whitney test respectively. We calculated 95% CIs for proportions. We performed data analysis using STATA16 (StataCorp, USA).

## Results

### Parental Consanguinity and Multiple Organ System Involvement Was Reported in Half of Cases

We recruited 183 cases from pediatric endocrinology clinics, predominantly from Turkey (56%), Pakistan (15%) and India (7%). Median age of diabetes onset was 7 years (IQR 3-11) and body mass index (BMI) z-score −0.3 (IQR −1.3 to −0.8). Among the cases, 91 (50%) participants reported parental consanguinity and 99 (54%) individuals exhibited additional features affecting 2 or more organ systems. Most affected systems were neurological (18.7%), musculoskeletal (13.2%), and eye (12.3%). Cohort characteristics (Table S2) and reported additional features (Table S3) are provided in the Supplementary Material (14).

### Monogenic Diabetes Was Diagnosed in a Third of Cases With At Least One Non-Autoimmune Clinical Feature

**Figure 1.**
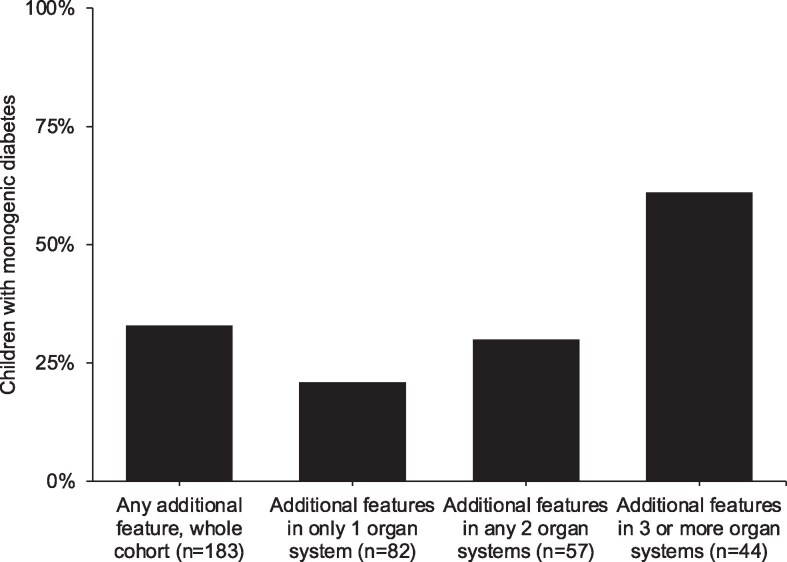
Percentage of children with diabetes of monogenic etiology in entire cohort (n = 183) and split by number of affected organ systems.

**Figure 2.**
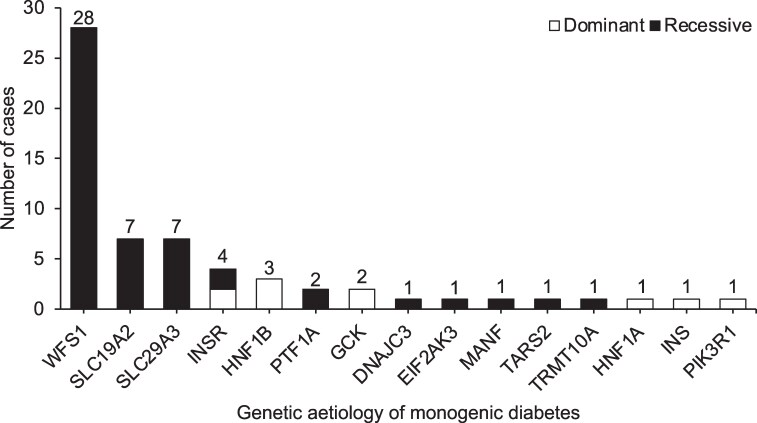
Genetic etiologies of monogenic diabetes identified in the syndromic diabetes cohort, split by pattern of inheritance.

Unselected testing of monogenic diabetes genes identified genetic cases in 33% (61/183) of children (Fig. 1). The proportion of monogenic cases was 28% in children with 1 affected organ system, 27% with 2 organ systems and 45% with 3 or more organ systems. This distribution was different from children without a pathogenic variant (53%, 32%, and 14% respectively, *P* = .000006) Recessive pathogenic variants were identified in 84% (51/61) and dominant variants in 16% (10/61). The most common genetic etiologies were *WFS1* (46%), *SLC19A2* (12%), and *SLC29A3* (12%) (Fig. 2, Table S4) (14). Five children (8%) had pathogenic variants that do not cause syndromic diabetes (*GCK* [n = 2], *HNF1A* [n = 1], *INS* [n = 1], and the *PTF1A* enhancer [n = 1]); thus, these additional features are likely to be coincidental and unrelated to the monogenic diabetes diagnosis.

### Multiple Organ System Involvement Is More Common in Monogenic Cases and Half Have Recognized Syndromic Manifestations

Monogenic and non-monogenic cases showed similar age at diagnosis, BMI z-score, glycated hemoglobin (HbA1c), and familial diabetes history (Table 1). However, monogenic cases had higher parental consanguinity (62% vs 19%, *P* = .012) and multiple organ system involvement (2 or more systems) compared to non-monogenic cases (72% vs 46% *P* = .000006; Table 1, Fig. S1) (14). Of cases with pathogenic variants in syndromic diabetes genes (n = 56), 59% (33/56) exhibited recognized clinical features of their syndrome (Table S5) (14). Limiting analysis to Wolfram syndrome (n = 28) yielded similar results (17/28, 60%). Among monogenic cases, abnormalities in eye (68% vs 31%, *P* = 1 × 10^−7^), ear, nose and throat (ENT) (64% vs 36%, *P* = 1 × 10^−6^), and renal (63% vs 37%, *P* = .005) systems were more common (Fig. S2) (14). This was likely driven by Wolfram syndrome (*WFS1*), since when those cases removed, no features were significantly enriched in monogenic cases (Fig. S3) (14).

**Table 1.** Comparison of characteristics between children with and without pathogenic variants in known monogenic diabetes genes in the syndromic diabetes cohort

	Children with monogenic etiology	Children without monogenic etiology	*P* value
n	61	122	
**Age at diagnosis, y**	6 (2-10)	7.4 (4-11)	.17
**Duration of diabetes, y**	4 (1.7-9.4)	2.4 (0.8-5.7)	.012
**Age at recruitment, y**	12.8 (8.9-15.9)	11.8 (8.2-15.5)	.39
**BMI Z-score**	−0.41 (−1.7 to 0.72)	−0.076 (−1.3 to 0.96)	.30
**Sex (female)**	29 (48%)	66 (54%)	.25
**Height, m**	1.36 (1.2-1.51)	1.3 (1.03-1.48)	.35
**Parental diabetes**	10 (16%)	14 (11%)	.24
**Current insulin treatment**	51 (85%)	109 (89%)	.27
**HbA1c**			.42
%	8.8 (7.3-11)	9.5 (7.5-11)	
mmol/mol	73 (56-97)	80 (58-97)	
**Self-reported consanguinity**	38 (62%)	23 (19%)	.**012**
**Number of affected organ systems**			.**000006**
1	17 (28%)	65 (53%)	
2	16 (27%)	40 (32%)	
>3	28 (45%)	17 (14%)	

Median (interquartile range) for continuous variables and number (percentage) for categorical data. Bold indicates clinically significant *P* values.

### Islet-Autoantibody-Negativity and Low T1DGRS Help Identify Cases for Genetic Testing

**Figure 3.**
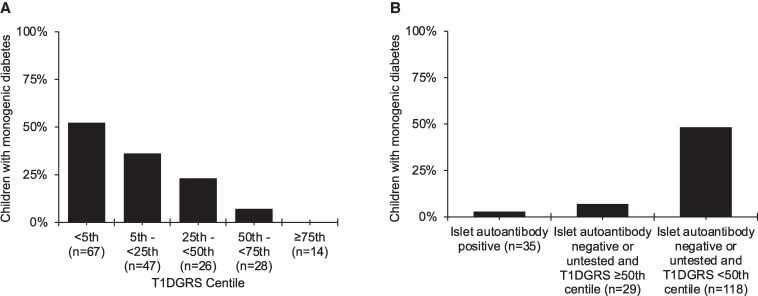
Utility of T1DGRS and islet autoantibodies in selecting children for monogenic diabetes testing. A, Percentage of monogenic diabetes in our study cohort (n = 183) across T1DGRS centile based on gold standard T1D population from Wellcome Trust Case Control Consortium (WTCCC) (n = 1963) (13). B, Percentage of monogenic diabetes in our study cohort (n = 183) by combination of islet autoantibody status and T1DGRS centiles.

Higher rates of monogenic diabetes were observed in the islet-autoantibody-negative cohort compared to the islet-autoantibody-positive cohort (48% [34/71] vs 3% [1/35], *P* = 1 × 10^−6^). In participants with T1DGRS (n = 182), higher rates of monogenic diabetes were identified in the low T1DGRS cohort (<50th centile of the T1D population) compared to high T1DGRS (≥50th centile) (58% vs 3%, *P* = 1 × 10^−6^, Fig. 3A). Limiting T1DGRS analysis to single ancestry (Turkish) yielded similar results (*P* = .004). When used in combination, children with low T1DGRS and negative/untested autoantibodies had higher rates of monogenic diabetes compared to high T1DGRS and negative/untested autoantibodies, and positive islet-autoantibodies (48% [57/118] vs 7% [2/29] vs 3%, [1/35], *P* < .0001, Fig. 3B). When restricting testing to negative/untested antibodies status and low T1DGRS, genetic testing was reduced by 35% (64/183) while retaining a 95% (57/60) sensitivity.

## Discussion

Using the largest cohort of its kind, our findings reveal a high proportion (33%) of monogenic diabetes among children with at least one non-autoimmune feature. This is higher than previous clinically selected MODY studies across populations with higher and lower rates of consanguinity (∼10%-20%) (3, 5, 18, 19). Among the cases, 84% have recessively inherited pathogenic variants, with *WFS1*, *SLC19A2*, and *SLC29A3* being most common. This differs from studies in England and France, where *HNF1B* and MIDD are the most common syndromic diabetes etiologies (3, 5), likely reflecting the highly consanguineous nature of our cohort. Further, 8% of cases have genetic etiologies that do not cause diabetes syndromes, highlighting the importance of performing a comprehensive monogenic panel for these children that includes syndromic, non-syndromic (eg, MODY), and dominant and recessive diabetes genes.

Our study supports the utility of islet-autoantibody status and T1DGRS in selecting syndromic patients for genetic testing. Previous studies have demonstrated the use of these biomarkers in selection for MODY testing (15, 20). We found that cases with negative islet-autoantibodies and a low T1DGRS were enriched for monogenic diabetes. T1DGRS offers an added advantage where it can be utilized if islet-autoantibody status is negative or untested by excluding those with high T1DGRS from genetic testing or explaining diabetes etiology when monogenic testing is negative (20). In our cohort, a case with a biallelic *SLC29A3* mutation had positive islet-autoantibodies and low T1DGRS. As *SLC29A3* variants cause an autoinflammatory H-syndrome phenotype (21), the presence of autoantibodies suggests this may not be coincidental but part of the autoinflammatory process.

Our study suggests that all children with diabetes and non-autoimmune features should be considered for monogenic diabetes testing, irrespective of number or type of feature. Diabetes-related features (age at diagnosis, BMI, treatment, and HbA1c) cannot reliably differentiate monogenic from non-monogenic cases in this cohort. Although monogenic cases are more likely to have additional features affecting multiple organ systems, 28% of cases had one affected organ system. Relying on specific clinical features may be less useful in consanguineous populations where there are higher prevalences of recessively inherited non-diabetes genetic syndromes. Although this concept of targeting genetic screening in those with pleiomorphic clinical signs is well categorized in classical clinical genetics, the continual discovery of novel monogenic diabetes genes and their variable clinical expressivity poses increasing diagnostic challenges for health care professionals. This along with a higher probability of monogenic etiology, we recommend a strategy that considers genetic testing of children with diabetes and any non-autoimmune feature. This approach is also easily implemented in routine clinical practice worldwide.

Our study has limitations. Our cohort originated from highly consanguineous populations, which limits extrapolation to non-consanguineous populations. However, due to global migration, clinicians working in outbred population are now increasingly providing care for individuals with parental consanguinity, making it relevant for wider health care professionals. The relatively small cohort size means our estimate of monogenic diabetes is less precise and may change when a similar study is performed with a larger cohort. However, given the rarity of the disease and the consanguineous population studied, our cohort remains the largest of its kind. This strength offers a valuable foundation for future research into syndromic diabetes. Not all islet-autoantibody testing occurred in a central laboratory, and thus processing methods may not be standardized. We used the targeted next-generation sequencing panel which limits identification of novel causal genes. Future studies are needed with a non-targeted approach to enable further novel gene discovery. It is possible that recognizable clinical features were present but not reported at the time of recruitment. Finally, the T1DGRS centiles were derived from a European cohort (17). T1DGRS is lower in non-European individuals with T1D, which may reduce the utility of T1DGRS in our cohort. Using ancestry-specific controls would improve validity here.

In conclusion, children with diabetes and at least one non-autoimmune extra-pancreatic feature from populations with high consanguinity have a high probability of monogenic diabetes and should be considered for targeted comprehensive monogenic diabetes testing.

## Data Availability

The clinical data generated and/or analyzed as part of this study are not publicly available because of patient confidentiality and due to the ethics of the study but are available from the corresponding authors upon reasonable request. Clinical information on individual patients with specific monogenic diabetes mutations is available upon request in order to assist other laboratories and clinicians with variant interpretation and genetic diagnosis of their patients.

## Abbreviations

BMI: body mass index
HbA1c: glycated hemoglobin
MIDD: maternally-inherited diabetes and deafness
MODY: maturity-onset diabetes of the young
T1D: type 1 diabetes
T1DGRS: type 1 diabetes genetic risk score
